# Hypoxia-Inducible Factors Modulate the Stemness and Malignancy of Colon Cancer Cells by Playing Opposite Roles in Canonical Wnt Signaling

**DOI:** 10.1371/journal.pone.0112580

**Published:** 2014-11-14

**Authors:** Paula Santoyo-Ramos, María Likhatcheva, Eduardo A. García-Zepeda, M. Cristina Castañeda-Patlán, Martha Robles-Flores

**Affiliations:** Department of Biochemistry, Faculty of Medicine, and Biomedical Research Institute, Universidad Nacional Autónoma de México (UNAM), Mexico City, Mexico; Western University, Canada

## Abstract

This study examined the role played by hypoxia-inducible factors (HIFs) in malignant phenotype maintenance and canonical Wnt signaling. Under normoxia, we determined that both HIF-1α and HIF-2α are expressed in human colon cancer cells but not in their non-malignant counterparts. The stable knockdown of HIF-1α or HIF-2α expression induced negative effects on the malignant phenotype of colon cancer cells, with lactate production, the rate of apoptosis, migration, CXCR4-mediated chemotaxis, and tumorigenic activity all being significantly affected by HIF knockdown and with HIF-1α depletion exerting greater effects. Knockdown of these two HIF transcripts induced different and even opposite effects on β-catenin transcriptional activity in colon cancer cells with different genetic Wnt signaling pathways. In SW480 cells, HIF-2α knockdown did not affect β-catenin levels, increasing the transcriptional activity of β-catenin by inducing its nuclear accumulation, whereas HIF-1α silencing negatively affected the stability and transcriptional activity of β-catenin, inducing its exit from the nuclei and its recruitment to the cell membrane by E-cadherin. In addition, although HIF-1α depletion induced a reversal of the epithelial-to-mesenchymal transition (EMT), HIF-2α silencing altered the expression of the stem cell markers CD44, Oct4, and CD24 and of the differentiation marker CK20 in the opposite direction as HIF-1α silencing. Remarkably, HIF-2α knockdown also enhanced β-catenin transcriptional activity under hypoxia in cells that displayed normal Wnt signaling, suggesting that the gene negatively modulates canonical Wnt signaling in colon cancer cells. Taken together, our results indicate that HIFs play opposing roles in canonical Wnt signaling and are essential for the stemness and malignancy maintenance of colon cancer cells.

## Introduction

Wnt signaling has been well-characterized as one of the most important contributors to tumorigenesis in many types of solid tumors. Aberrant canonical Wnt signaling is known to contribute to early progression in the majority of colorectal cancers. Indeed, a great amount of experimental evidence has shown that mutations in the adenomatous polyposis coli (APC) gene act as gatekeepers in the molecular pathogenesis of the majority of sporadic and hereditary forms of colorectal carcinoma [1], [2]. The Wnt pathway has also been demonstrated to play an important role in the development and regulation of adult stem cell systems, and canonical Wnt signaling supports the formation and maintenance of both stem and cancer stem cells (CSC) [3].

Canonical Wnt signaling operates through the regulation of the phosphorylation and degradation of the transcription co-activator β-catenin. Without stimulation by Wnt, β-catenin is assembled into the so-called destruction complex, in which APC plays a central role, and this complex also includes axin, GSK-3β and Casein kinase 1. This complex directs a series of phosphorylation events in β-catenin that make it a target for ubiquitination and subsequent proteolysis via the proteasome [4]. Stimulation by Wnt leads to the inhibition of β-catenin breakdown, allowing β-catenin to accumulate, enter the nucleus, and activate Wnt target genes such as *CYCLIN D1* and *C-MYC* proto-oncogenes, which promote the entry of the cell into the S phase of the cell cycle [5].

Tumor hypoxia and the critical mediators of the cellular oxygen signaling pathway, namely the hypoxia-inducible factors (HIFs), are known to regulate multiple steps of tumorigenesis and are typically associated with changes in metabolism, neo-vascularization, invasion, metastasis, drug resistance, and ultimately poor clinical outcomes [6]. HIFs are heterodimeric transcription factors consisting of HIF-α and HIF-β (or ARNT) that are expressed constitutively at the transcriptional and translational levels. HIF-1α and HIF-2α (also known as EPAS1) are the two best-studied members of the HIF-α family. Under normoxic conditions, the HIF-α subunits are hydroxylated at key proline residues, which allows them to be recognized by the von Hippel-Lindau (pVHL) tumor suppressor, the substrate recognition component of an E3 ubiquitin ligase complex that targets HIF-α for proteasomal degradation. Hypoxic signaling stabilizes HIF-α by inhibiting prolyl hydroxylation, and in turn ubiquitin proteasomal degradation, making HIF-α capable of dimerizing with ARNT, binding to the hypoxia-responsive DNA element, and recruiting the transcription coactivator p300/CBP for the transcriptional activation of a host of hypoxia-responsive genes [7].

Given the structural similarities of HIF-1α and HIF-2α, they were thought to act redundantly in the cellular response to hypoxia. However, a growing body of evidence indicates that HIF-1α and HIF-2α induce the expression of different sets of genes. Although HIF-1α and HIF-2α have shared targets such as vascular endothelial growth factor (VEGF), they also regulate unique gene targets; HIF-1α regulates glycolytic enzymes [8] and HIF-2α activates the stem cell factor Oct4 [9]. Consistent with this finding, Imamura et al. identified distinct sets of HIF-1α and HIF-2α target genes in SW480 colon cancer cells by cDNA microarray analysis [10].

Crosstalk has been reported between canonical Wnt signaling and HIF signaling in tumor progression and metastasis. However, the molecular mechanisms involved in this crosstalk remain poorly understood. Several reports have indicated that the activities of the transcription factors regulated by hypoxia play an important role in regulating stem cell quiescence [9] and that HIF-1α-mediated Wnt activation promotes the maintenance of stem cell activity [11]. Mazumdar et al. showed that HIF-1α modulates Wnt/β-catenin signaling in hypoxic embryonic stem cells by enhancing β-catenin activation and the expression of the downstream effectors LEF-1 and TCF-1 [11]. In addition, there is experimental evidence supporting the hypothesis that both hypoxic conditions and canonical Wnt activation are involved in triggering an EMT program in many cancer cells [12], [13]. Furthermore, crosstalk has been shown to exist between canonical Wnt signaling and HIF signaling in tumor progression and metastasis via the synergistic interaction of the Wnt target gene *c-MYC* with HIF-1α [14], [15]. In this regard, although normal physiological HIF-1α responses in non-malignant cells can inhibit the activity of normal c-Myc, paradoxically, the deregulated expression of oncogenic c-Myc that exists in many cancers such as colorectal carcinoma works together with HIF-1α to confer the tumor metabolic phenotype described as the Warburg effect or aerobic glycolysis as well as to promote angiogenesis [14].

Current knowledge of the homeostatic mechanisms that regulate the epithelial colon stemness and malignancy remains incomplete, preventing significant advancements in the treatment of colon carcinoma. This study examined the effects of stable HIF-1α and HIF-2α siRNA knockdown in malignant phenotype maintenance and in the transcriptional activity mediated by β-catenin. Our results indicate that although both HIF-1α and HIF-2α are essential for stemness and malignancy maintenance, these two proteins exert different effects and play opposing roles in canonical Wnt signaling.

## Materials and Methods

### Reagents and antibodies

The following antibodies were used in these experiments: allophycocyanin-conjugated mouse anti-CD44, mouse anti-CD44, and mouse anti-CD24 from BD Biosciences (San Jose, CA, USA); phycoerythrin (PE)-conjugated mouse anti-CD133 from Miltenyi Biotec (Auburn, CA, USA); mouse anti-HIF-1α, mouse anti-HIF-2α, rabbit anti-Oct4, Alexa 647-conjugated rabbit anti-mouse, Cy3-conjugated goat anti-rabbit, and mouse anti-lamin A/C from Millipore (Billerica, MA); rabbit anti-β-tubulin from Cell Signaling Technology (Danvers, MA, USA); biotin-conjugated mouse anti-CXCR4 from R&D Systems (Minneapolis, MN, USA); mouse anti-β-catenin, mouse anti-cytokeratin 20 (CK20), rabbit anti-E-cadherin, rabbit anti-Snail 1, mouse anti-vimentin, rabbit anti-GSK-3β, and goat anti-(p-Ser9)-GSK-3β from Santa Cruz Biotechnology (Santa Cruz, CA, USA); Alexa647-conjugated goat anti-rabbit from Molecular Probes, Inc., (Eugene, OR, USA); and allophycocyanin-conjugated streptavidin from Biolegend (San Diego, CA, USA). Mouse IgG1 and IgG2b from US Biologicals (Massachusetts, MA, USA) were used at the same concentration as the primary antibody, as was the isotype matched control in the immunofluorescent staining and flow cytometry experiments. The Annexin V FLUOS Staining Kit from Roche Applied Science (Mannheim, Germany) was used to detect apoptotic and necrotic cells. The puromycin antibiotic and L-lactate dehydrogenase were obtained from Sigma–Aldrich (St. Louis, MO, USA). A growth factor-reduced Matrigel matrix was purchased from BD Biosciences. All other chemicals were of reagent grade.

### Plasmids

The pTOPFlash and pFOPFlash reporter plasmids were obtained from Upstate Biotechnology (Lake Placid, NY, USA). The HRE-Luc reporter was obtained from Addgene (Plasmid 26731: HRE-luciferase), a non-profit organization dedicated to facilitating plasmid sharing among scientists. The control plasmid encoding a scrambled shRNA sequence was obtained from Santa Cruz Biotechnology. The control (void plasmid pSuper), HIF-1α, and HIF-2α RNAi plasmids were generous gifts from Dr. Daniel Chung, and their construction and effectiveness were described in a previous report [10].

### Cell culture

All cancer cell lines and the non-malignant 112CoN cell line used here were purchased from American Type Culture Collection (ATCC; Manassas, VA, USA) and cultured as previously described [16]. All of these cell lines were authenticated in January 2012 by Short Tandem Repeat DNA profiling performed at the Instituto Nacional de Medicina Genómica (INMEGEN) in Mexico City.

### Western blotting

Samples of protein (50 µg) were separated by 10% sodium dodecyl sulfate–polyacrylamide gel electrophoresis (SDS-PAGE) followed by electrophoretic transfer onto nitrocellulose membranes (Bio-Rad, Hercules, CA, USA) as described in a previous report [14]. An actin antibody was used to control for equal loading.

### Immunoprecipitation

The cells were washed and homogenized in ice-cold pH 7.5 lysis buffer containing 50 mM Tris, 150 mM NaCl, 0.5% Triton X-100 and a mixture of protease inhibitors and protein phosphatase inhibitors. The protein concentration in the supernatant was measured using a detergent-compatible protein assay (Bio-Rad). Aliquots of these extracts (1 mg/ml) were incubated overnight at 4°C with 2 µg/ml primary antibody with gentle shaking. Then, 25 µl of protein A-sepharose (30%, Calbiochem) was added and incubated for 2 h. The immune complexes were then washed twice with buffer A (50 mM Tris-HCl and 0.6 M NaCl, pH 8.3) supplemented with 0.1 mg/ml trypsin inhibitor and 1 mM PMSF, and once with buffer B (50 mM Tris HCl and 0.15 M NaCl, pH 7.5) containing protease and phosphatase inhibitors.

### HIF-1α or HIF-2α knockdown

To induce the stable silencing of HIF-1α or HIF-2α, the cells were transfected with the pSuper HIF-1α or HIF-2α RNAi plasmid, which were constructed and analyzed by Dr. Daniel Chung as described in a previous report [10] or with the control plasmid (encoding a scrambled shRNA sequence or pSuper void plasmid) using Lipofectamine 2000. To generate stable transfections, the cells were transfected with either 1 µg of the control plasmid or 1 µg of pSuper HIF-1α RNAi or HIF-2α RNAi plasmids. Stable transfectants were selected with 3 µg/ml puromycin (Sigma) for four weeks, and the clones were selected and screened for HIF-1α or HIF-2α silencing by flow cytometry.

### FACS analysis

The cells were detached and dissociated in 10 mM EDTA solution. The cell suspension was washed, resuspended in PBS supplemented with 4% fetal calf serum (FCS) (staining buffer), stained with the corresponding primary antibody, and then incubated with the secondary antibody. Cells stained with the secondary antibody alone were used as a negative control. For nuclear staining, nuclei were purified from the cell samples using a Nuclei Isolation Kit (Sigma–Aldrich) according to the manufacturer’s instructions. The nuclei were washed, fixed, permeabilized, blocked, and labeled with anti β-catenin and Alexa 647-conjugated goat anti-mouse antibody in staining buffer. As a negative control, nuclei maintained in separate tubes were probed in parallel with Alexa 647-conjugated goat anti-mouse antibody. After a final wash, the nuclei were fixed and analyzed by flow cytometry.

### Lactate measurement assay

The amount of lactate the cancer cells secreted into the culture medium was measured using an enzymatic assay using L-lactate dehydrogenase (Sigma). In this assay, the lactate secreted into the culture medium sample is reduced to pyruvate and NADH in the presence of lactate dehydrogenase (LDH) (Sigma) and excess NAD. The amount of NADH formed in the reaction, measured by the change in absorbance at 340 nm, is proportional to the concentration of lactate present in the sample. To avoid interference with the LDH that may already be present in the serum used to supplement the culture medium, the samples were subjected to deproteinization with 8% trichloroacetic acid (TCA) to render them protein-free prior to the assay.

### Apoptosis

SW480 control or HIF-1α or HIF-2α-silenced cells were seeded onto 24-well plates at a density of 1.5×10^5^ cells per well. Twenty-four hours after seeding, the cells were incubated in the absence or presence of the apoptosis inducer H_2_O_2_ (1 mM) for 12 h. Apoptosis was measured by flow cytometry using the Annexin V FITC kit (Roche) as recommended by the manufacturer’s instructions. Necrosis was measured by propidium iodide (PI) permeability in the absence of detergent. The percentage of apoptotic cells was determined by flow cytometry.

### Wound-healing assay

Control or HIF-1α- or HIF-2α-silenced SW480 cells were seeded to confluence onto poly-L-lysine coated slides in DMEM F12 supplemented with 5% FBS. After 24 h, a scratch was produced using a sterile pipette tip. The cultures were washed with PBS. At this time point (t = 0 h), the wound margins were photographed. The cells were then cultured in medium supplemented with 0.05% FBS for up to 72 h, and the wound margins were photographed at different time points.

### Migration assay

The chemotactic response to the stromal cell-derived factor-1α (SDF-1α) of the HIF-1α and HIF-2α-silenced and control cells was assessed using Boyden chambers (3 µm pore size). A total of 1×10^5^ cells were added to the upper part of the chamber, and the lower part of the chamber contained SDF-1α (200 ng/ml in 0.05% FBS). To obtain the absolute numbers of migratory cells, flow cytometric counts for each sample were obtained for a constant, predetermined volume and then compared with duplicate flow cytometric counts obtained from the control wells.

### Xenograft tumor model

Selected clones of stable control-, HIF-1α-, or HIF-2α-transfected cells were selected and screened by flow cytometry to determine the HIF-1α and HIF-2α silencing efficiency in comparison with the control cells. The cells displaying the highest knockdown efficiency were cultured and used for xenotransplantation into immunocompromised mice. For each injection site, 1×10^6^ HIF-1α- or HIF-2α-silenced cells were resuspended in a high concentration of Matrigel (BD Biosciences), diluted in PBS to a final concentration of 50% and subcutaneously (s.c.) injected into the flanks of six-week-old nude mice (n = 5). Each mouse was injected in the upper right flank with control cells, into the bottom right flank with HIF-1α-silenced cells, and into the bottom left flank with HIF-2α-silenced cells.

For xenotransplants using CD44^−^/CD133^−^ or CD44^+^ subpopulations, cells were obtained from SW480 cells stably transfected with the control pSuper plasmid or with pSuper HIF-1α or HIF-2α RNAi by FACS cell sorting. The purified subpopulations for each condition were collected by trypsinization. Then, 1×10^4^ cells per injection site were resuspended in growth factor-reduced Matrigel matrix, diluted in PBS to a final concentration of 50% and s.c. injected into the flanks of six-week-old nude mice. Each mouse (n = 5 for each condition) was injected into the right flank with CD44^−^/CD133^−^ cells and into the left flank with CD44^+^ cells purified from each condition (control, HIF-1α knockdown, or HIF-2α knockdown). Four weeks (for RKO or SW480-derived cells) or two weeks (for SW620-derived cells) after inoculation, the animals were euthanized and the tumors were removed and weighed.

### Transfection and luciferase reporter gene assay

The cells were seeded on 24-well plates at a density of 1.2–1.8×10^5^ cells per well. Twenty-four hours after seeding, the cells were placed in serum-free medium and transfected with 1 µg of a reporter plasmid (pTOPFlash) or control plasmid (pFOPFlash) and with 0.05 µg of the pRL luciferase plasmid or CMV-GFP plasmid (transfection control). The luciferase reporter activity in the cell lysates was measured 24 h after transfection using the Dual Luciferase Assay kit (Promega, Madison, WI, USA). The activity was normalized with respect to the activity of *Renilla* luciferase or with respect to the protein content in each sample.

### Immunofluorescence analysis

SW480 control or HIF-1α- or HIF-2α knockdown cells were grown on coverslips. The cells were fixed, permeabilized and co-immunostained with antibodies against β-catenin, E-cadherin, vimentin and Snail 1. The fluorescence was analyzed by laser confocal microscopy as described in a previous report [16]. β-catenin and vimentin were visualized with Alexa 647-conjugated goat anti-mouse antibody, and E-cadherin and Snail 1 were visualized with Cy3-conjugated goat anti-rabbit antibody. The cell fluorescence was imaged using a confocal microscope (Leica TCS SP5) with a krypton argon laser. A control sample of cells was stained with the secondary antibody only to confirm that no fluorescence signal was detected from these cells.

### Ethics statement

All of the animals were handled in strict accordance with good animal practices as defined by the Animal Experimental Bio-Ethics Guidelines of the Instituto Nacional de Ciencias Médicas y Nutrición Salvador Zubirán, México. In addition, all animal studies were approved by the Animal Experimental Bioethics Committee of the Faculty of Medicine, Universidad Nacional Autónoma de México. When indicated, the mice were euthanized with CO_2_.

### Statistical analysis

The data are expressed as the mean ± standard error of the mean (SEM). Statistical data analysis was performed using Student’s *t* test or a one-way-ANOVA with Tukey’s multiple comparison test. A value of p<0.05 was considered statistically significant.

## Results

### HIF-1α and HIF-2α are expressed in colon cancer cells but not in non-malignant cells under normoxic conditions

**Figure 1:**
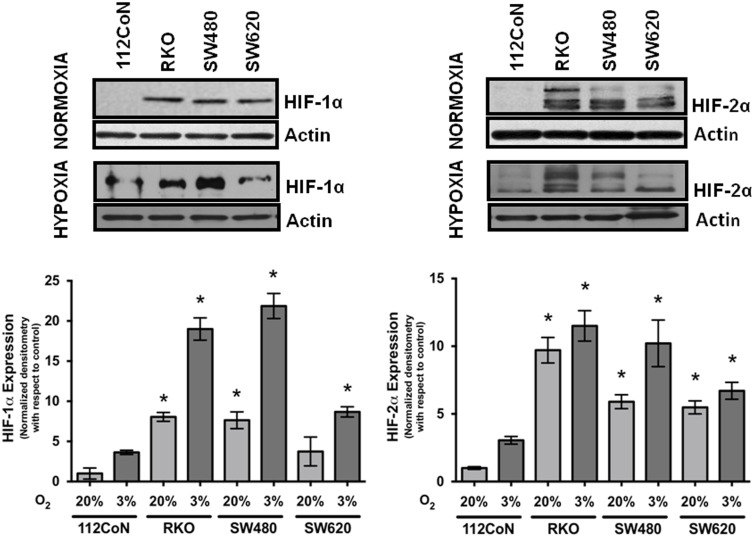
HIF-1α and HIF-2α are co-expressed in colon cancer cells but not in non-malignant cells under normoxic conditions. Normal colon (112CoN) or colon cancer cells were cultured under normoxia (20% O_2_) or hypoxia (3% O_2_) for 12 h. Total cell extracts of the colon cell lines were prepared, and the samples were subjected to 10% SDS-PAGE and transferred onto nitrocellulose membranes. An immunoblot analysis was performed using anti-HIF-1α or anti-HIF-2α antibodies as indicated in the figure, and developed using a horseradish peroxidase-conjugated second antibody. Actin antibody was used to control for equal loading. A densitometric analysis was performed to estimate the levels of HIF-1α and HIF-2α expression, which were normalized to the corresponding expression levels in non-malignant 112CoN cells. All of the assays were performed in triplicate, and the data represent the means ± SEM from at least three independent assays. *p<0.05.

Hypoxia is a common condition observed in a wide range of solid tumors, and HIF over-expression is frequently associated with metastasis and poor clinical outcomes. *In vivo*, hypoxia is also likely to be a functional component of a normal stem cell niche. However, the importance of hypoxia in CSC maintenance remains largely unknown [17]. Because high HIF expression has been detected in tumor cells in the absence of hypoxia, we compared the expression of these factors in cultured colon cancer cells under normoxic and hypoxic conditions with cultured non-malignant 112CoN cells that were incubated under the same conditions by western blot. The results shown in Figure 1 indicate that as expected, hypoxia (3% O_2_) induced the expression of both HIF-1α and HIF2-α in both normal (112CoN) and cancerous cells; however, under normoxic culture conditions (20% O_2_), only the colon cancer cells co-expressed both HIF-1α and HIF-2α, while the non-malignant 112CoN cells did not express these factors under normoxic conditions.

### Stable knockdown of HIF-1α but not HIF-2α decreases lactate production by colon cancer cells under normoxic conditions

**Figure 2:**
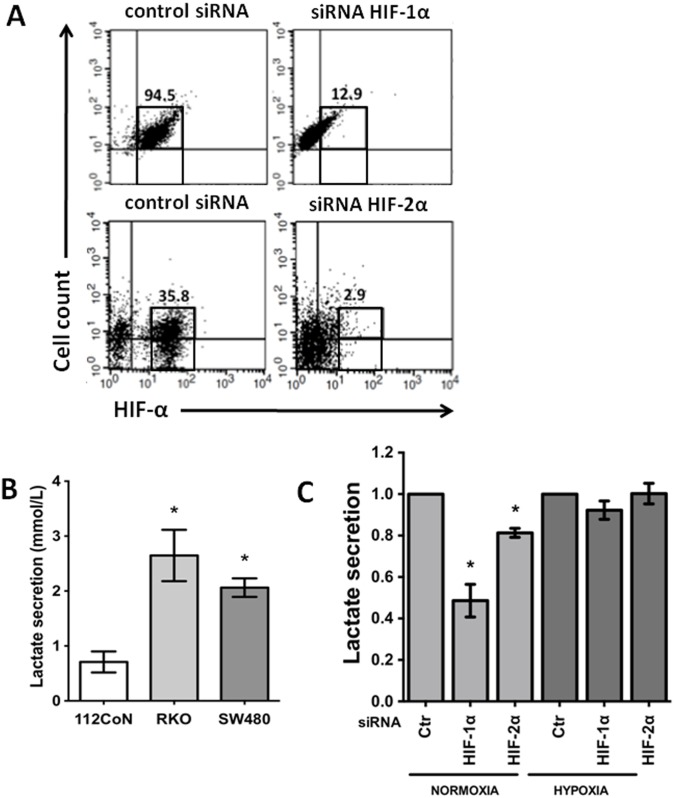
Stable knockdown of HIF-1α but not HIF-2α decreases lactate production by colon cancer cells. **A)**. Stable HIF-1α- or HIF-2α-knockdown or control (scrambled shRNA plasmid) transfectants were obtained as described in the “Materials and Methods”. Selected clones were screened by flow cytometry to assess the silencing of HIF-1α and HIF-2α in comparison with the control cells. **B)**. Lactate secretion was significantly increased in colorectal cancer cell lines compared with non-malignant 112CoN cells. **C)**. Changes in lactate secretion by HIF-1α- or HIF-2α- silenced SW480 cells in comparison with the control cells (Ctr in the figure) cultured under normoxia (20% O_2_) or hypoxia (3% O_2_) for 24 h. L-lactate was determined by an LDH enzymatic assay as described in the “Materials and Methods”. All of the assays were performed in triplicate, and the data represent the means ± SEM from at least three independent assays. *: p<0.05.

To understand the roles played by HIFs in cancer phenotype maintenance and their possible interaction with canonical Wnt signaling, we examined the effects of the stable knockdown of HIF-1α and HIF-2α by siRNA in SW480 cells, which exhibit constitutively active canonical Wnt signaling. The plasmids used in this study were constructed and previously successfully probed by Dr. Daniel C. Chung [10], who kindly donated the plasmids to us. SW480 cells were transfected with the control scrambled shRNA plasmid, pSuper HIF-1α RNAi, or pSuper HIF-2α RNAi. Stable transfectants were selected using an antibiotic for four weeks, and the clones were selected and screened by flow cytometry for HIF-1α and HIF-2α silencing in comparison with the control cells. Stable transfectants exhibiting a decrease of at least 85% in HIF-1α expression and a decrease of at least 90% in HIF-2α expression were obtained and selected by flow cytometry as shown in Figure 2A. Cancer cells exhibit increased glycolysis and lactate production and decreased O_2_ consumption compared with non-transformed cells, a phenomenon known as the Warburg effect [18]. Consistent with this effect, Figure 2B shows that a significant increase in lactate production was apparent in the SW480 and RKO colon cancer cells compared with the non-malignant 112CoN colon cells. We then examined the effects of the stable silencing of each HIF on lactate production by SW480 cancer cells under normoxia or hypoxia. As observed in Figure 2C, the stable knockdown of HIF-1α in SW480 cells decreased the lactate production by at least 50% compared with the control cells under normoxic conditions, whereas HIF-2α knockdown only led to a 20% decrease in lactate secretion compared with control cells under the same conditions (Figure 2B). After 12 h of exposure to acute hypoxia, the levels of HIF proteins were elevated, and the levels of lactate production recovered (Figure 2C). Thus, to avoid HIF overexpression, which could override the knockdown efficiency (shown in Figure S1) and make it difficult to dissect the role played by each HIF in the maintenance of the malignant phenotype, we decided to perform most analyses under normoxic conditions.

### Stable knockdown of HIF-1α or HIF-2α produced an increase in the basal or H_2_O_2_-induced apoptosis in SW480 colon cancer cells

**Figure 3:**
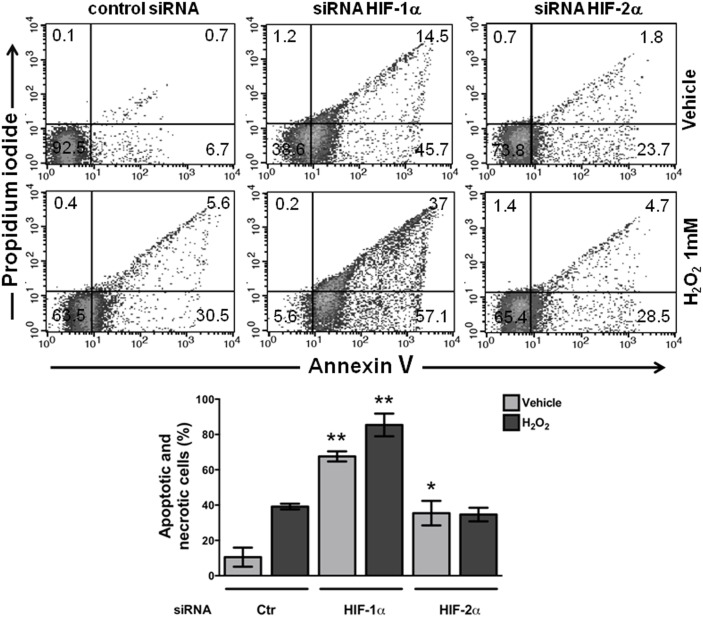
HIF knockdown increased the apoptosis of SW480 cells. Apoptosis was induced by culturing the HIF-silenced or control (transfected with scrambled shRNA plasmid and indicated in the figure as Ctr) cells in the absence or presence of 1 mM H_2_O_2_ for 12 h. SW480 cells were trypsinized, washed twice with PBS, and untreated (vehicle) or treated with H_2_O_2_ for 12 h. The cultures were stained with Annexin V and PI, as described in the “Materials and Methods”, and analyzed by flow cytometry. All of the assays were performed in triplicate (a representative histogram of the data is shown), and the graph presents the means ± SEM from three independent experiments; *: p<0.05; **: p<0.01.

It is well established that HIF-1α promotes cell survival and apoptosis resistance in several cell systems under hypoxia. We explored whether the blockade of HIF-1α or HIF-2α expression in cancer cells that express both factors under normoxia also affects cellular survival. We used hydrogen peroxide to induce acute apoptosis because it has been widely reported to be a potent inducer of apoptosis in cancer cells due to the production of severe oxidative stress. HIF-silenced SW480 cells were treated with 1 mM H_2_O_2_ for 12 h, and then the degree of cell apoptosis was determined by Annexin V-PI staining followed by flow cytometry analysis. Both apoptosis and necrosis were enhanced under normoxia as a result of HIF-1α or HIF-2α silencing compared with the control cells, even in the absence of the apoptosis inducer (upper part of Figure 3). However, the proportion of apoptotic and necrotic cells obtained as a result of HIF-1α silencing was greater than that induced by HIF-2α knockdown with respect to the controls, and this effect was more evident when the cells were treated for 12 h with 1 mM H_2_O_2_ (Figure 3). Thus, these results suggest that HIFs, particularly HIF-1α, are involved in the promotion of cell survival and resistance to apoptosis.

### Stable knockdown of HIF-1α or HIF-2α diminished the cell migration of SW480 cancer cells, but only HIF-1α knockdown severely affected CXCR4-mediated chemotaxis

**Figure 4:**
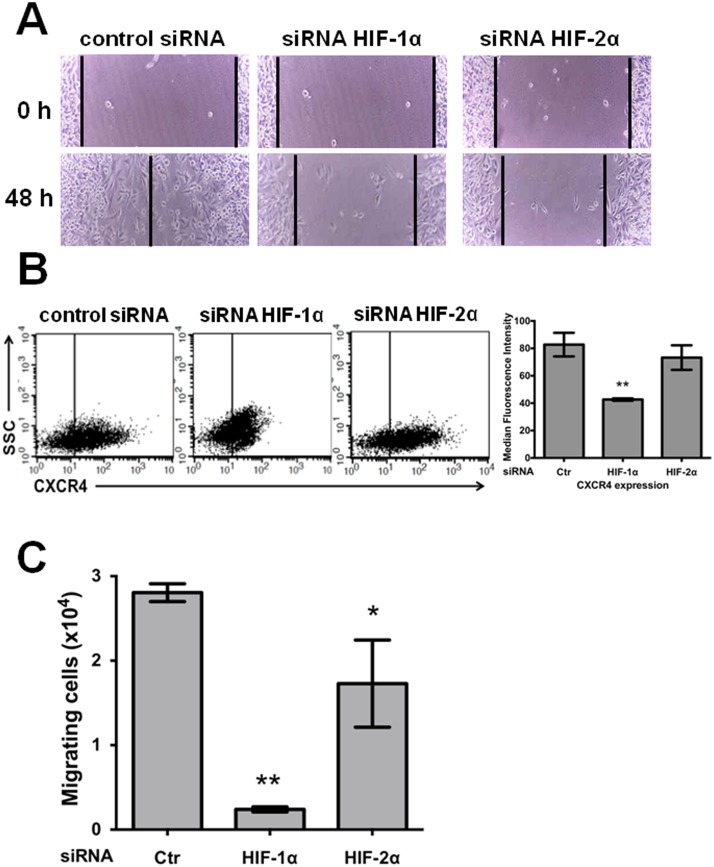
Stable knockdown of both HIF-1α and HIF-2α decreased the cell migration of SW480 cancer cells, but only HIF-1α knockdown severely affected CXCR4-mediated chemotaxis. **A)** A wound-healing assay was used to determine whether cellular migration is dependent on either HIF-1α or HIF-2α expression. SW480 knockdown and control (scrambled shRNA plasmid) cells were grown to confluence on 24-well tissue culture plates, and the wound-healing assay was performed as described in the “Material and Methods”. The scratched area was imaged immediately after wounding (time 0) and 48 h after wounding. These images are representative of three independent experiments. **B)** CXCR4 expression decreased as a result of HIF-1α knockdown. SW480 control or silenced cells, as indicated in the figure, were detached using EDTA and washed, and 1×10^5^ cells were incubated with mouse biotin-conjugated anti-CXCR4 and allophycocyanin-conjugated streptavidin antibody and examined by flow cytometry. The data show the median fluorescence intensities of CXCR4 expression and SSC (side-scattered light, proportional to cell granularity), and represent the mean values ± SEM from three independent experiments. *: p<0.05; **: p<0.01. **C)** Significant differences were observed in the SDF-1α-induced chemotactic response. The chemotactic response to SDF-1α of the HIF-1α- or HIF-2α-knockdown or control cells (Ctr in the figure) was assessed using Boyden chambers as described in the “Materials and Methods”. The cells were incubated for 48 h to allow for migration and recovered using EDTA. The results represent the means ± SEM from three experiments performed in duplicate. *: p<0.05; **: p<0.01.

We performed wound-healing assays to investigate the effects of these proteins on cell migration. Stable HIF-1α- or HIF-2α-silenced cells were grown under normoxia to confluency, and then scratches were made on the monolayer. Images of the scratches were captured at 0 and 48 h, and the scratch area was analyzed at these time points. As can be observed in Figure 4A, in comparison to the controls, migration was blocked by the knockdown of HIF-1α or HIF-2α (please see reference limit lines drawn on the pictures). In addition, the evaluation of cellular migration based on the chemotactic activity towards SDF-1α mediated by the CXCR4 receptor revealed that CXCR4 expression was decreased as a result of HIF-1α but not HIF-2α knockdown (Figure 4B). Consistent with this finding, the silencing of HIF-1α expression nearly abolished the SDF-1α- CXCR4-mediated migration of cancer cells through Transwell chambers, whereas the silencing of HIF-2α expression decreased cell migration by only 45% with respect to the controls (Figure 4C).

### Stable silencing of HIF-1α or HIF-2α decreased the in vivo tumorigenic activity of engrafted colon cancer cells

**Figure 5:**
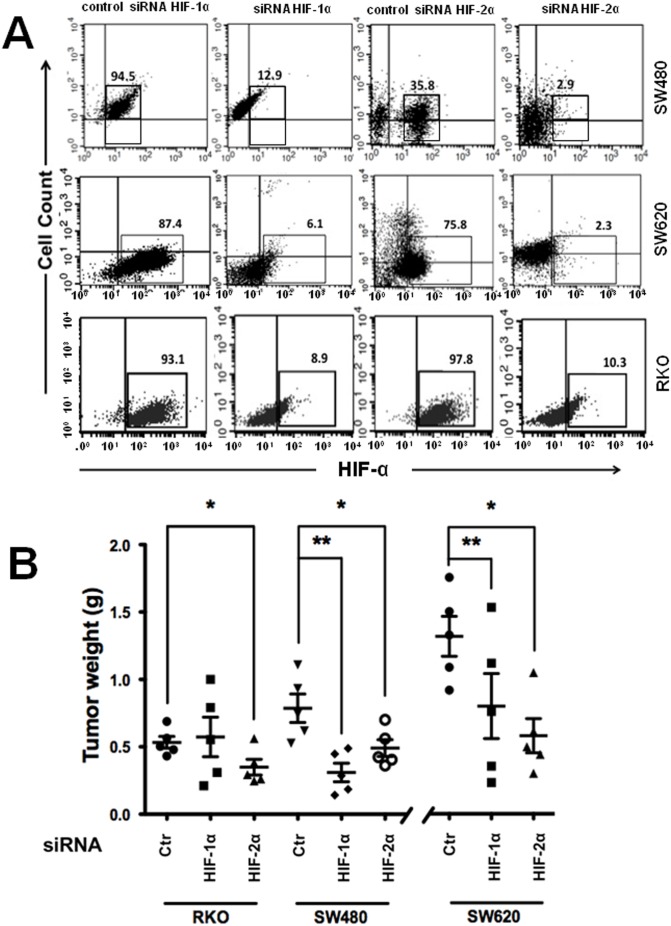
Stable silencing of HIF-1α or HIF-2α decreased the *in vivo* tumorigenic activity of engrafted colon cancer cells. **A)** Stable HIF-1α- or HIF-2α-knockdown or control transfectants were obtained as described in the “Materials and Methods” from the RKO, SW480, and SW620 colon cell lines. Selected clones were screened by flow cytometry to determine the silencing of HIF-1α and HIF-2α in comparison with the control cells. **B)** A total of 1×10^6^ stable control (Ctr in the figure) or HIF-1α- or HIF-2α-silenced RKO, SW480, or SW620 cells were s.c. injected into the dorsal region of healthy six-week-old female immunocompromised nude mice (n = 5 for each condition). Each mouse was injected in the upper right flank with control cells, in the bottom right flank with HIF-1α-silenced cells, and in the bottom left flank with HIF-2α-silenced cells. A total of 1×10^6^ cells in 100 µl of growth factor-reduced Matrigel were transplanted at each site. After four weeks (RKO and SW480 cells) or two weeks (SW620 cells), the mice were euthanized, and the tumors were removed and weighed. The data represent the means ± SEM from n = 5 for each experimental condition. *: p<0.05; **: p<0.01.

The effect of the stable siRNA-mediated knockdown of HIFs on the tumorigenic activity of colon cancer cells was studied using a xenograft model in immunocompromised nude mice. We examined this effect using representative colon cancer cell lines that exhibit different Wnt genetic contexts: human RKO malignant cells that have normal canonical Wnt signaling (these express the wild-type APC protein) and human SW480 malignant cells, which express a truncated version of APC and have constitutively active Wnt signaling. We also used SW620 cells, which are derived from a metastasis of the same tumor from which the SW480 cells were derived. Stable cells transfected with the control pSuper plasmid or with pSuper HIF-1α or HIF-2α were selected, and the silencing efficiency was determined by flow cytometry as shown in Figure 5A. The cells exhibiting the highest knockdown efficiency were selected by FACS, grown, and injected s.c. into the flanks of NOD/SCID mice. In these same animals, control cells were injected s.c. into the upper flank, and HIF-1α- and HIF-2α-silenced cells were injected s.c. into the bottom right flank and the bottom left flank, respectively. After four weeks (RKO and SW480 cells) or two weeks (SW620 cells), the mice were euthanized, and the tumors were removed and weighed. As can be observed in Figure 5B, the HIF-1α- or HIF-2α-depleted colon cancer cells exhibited a decrease in tumorigenic activity in the grafted mice compared with the controls; however, this trend was not observed with the RKO cells, in which HIF-1α silencing did not significantly inhibit tumorigenic activity. Note that the negative effects were more evident in colon cancer cells that exhibit altered Wnt signaling, particularly in the metastatic HIF-silenced SW620 cells, which as expected based on their more aggressive phenotype, exhibited larger xenografts in less time (two weeks instead of four) than the non-metastatic cells. Based on these results, we then examined the effects of the knockdown of HIF-1α or HIF-2α in CSCs.

**Figure 6:**
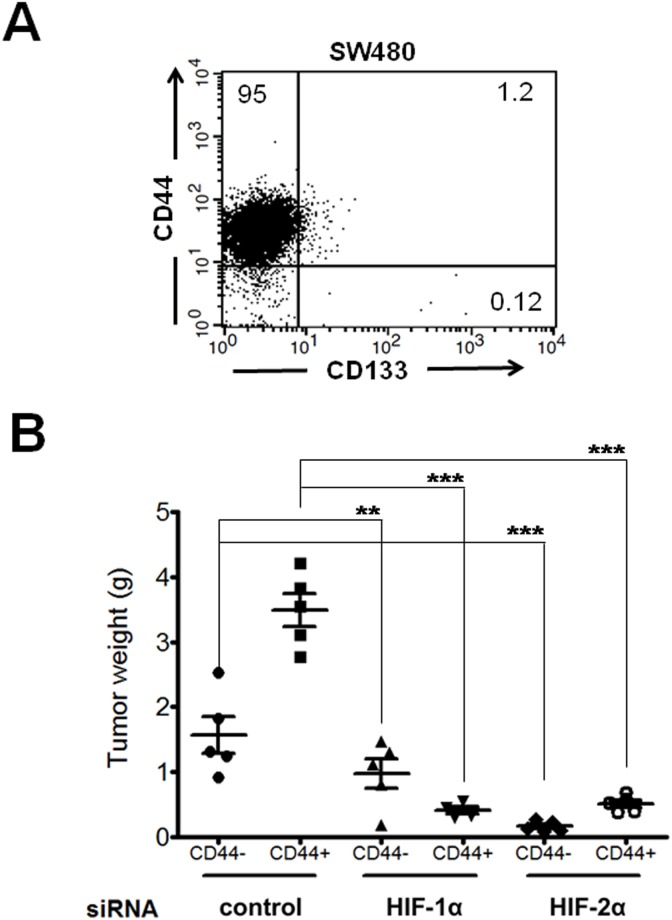
Decreased *in vivo* tumorigenic activity of FACS-purified CD44+ and CD44−/CD133− subpopulations from control or HIF-1α or HIF-2α-knockdown SW480 cells. **A)** Analysis of CD44/CD133 expression profiles in the SW480 colorectal cancer cell line. The cells were incubated in the presence of EDTA and washed, and 1×10^5^ cells were incubated with PE-conjugated anti-CD133 and allophycocyanin-conjugated anti-CD44 antibodies for 15 min at 4°C and examined using flow cytometry. **B)** The CD44^+^ (population corresponding to the upper left quadrant in panel A) or CD44^−^/CD133^−^ (population corresponding to the lower left quadrant) subpopulations from the stable control or HIF-1α- or HIF-2α-silenced cells were obtained by FACS cell sorting. The purified subpopulations were s.c. injected into the dorsal region of healthy six-week-old immunocompromised nude mice (n = 5 for each condition). Each mouse was injected in the right flank with the CD44^−^ cells and in the left flank with the CD44^+^ cells. A total of 1×10^4^ cells in 100 µl of growth factor- reduced Matrigel were transplanted at each site. Four weeks after inoculation, the animals were euthanized, and the tumors were removed and weighed. The data represent the means ± SEM for each experimental condition with n = 5. **: p<0.05; ***: p<0.01.

Several colorectal cancer CSC markers have been reported to date, including CD133, CD44, CD24, CD166, and Lgr-5 [19]. Because CD133 and CD44 have been widely validated as CSC markers in a variety of solid tumors, we analyzed their expression profile in SW480 cells by FACS. As shown in Figure 6A, these cells are highly enriched in CD44^+^ but do not express CD133, in agreement with previous reports [20]. The CD44^+^ (population corresponding to the upper left quadrant in Figure 6A) and CD44^−^/CD133^−^ (population corresponding to the lower left quadrant) cells were purified by FACS cell sorting from SW480 control or silenced cells, grown, and injected s.c. into the right and left flanks, respectively, of each mouse. After five weeks, the mice were euthanized, and the tumors were removed and weighed. The results presented in Figure 6B show that all of the mice engrafted with the CD44^+^ or CD44^−^/CD133^−^ cell populations obtained from the control cells produced tumors at the time of euthanasia and that the CD44^+^ cells were more tumorigenic. Remarkably, all of the CD44^+^ or CD44^−^/CD133^−^ engrafted cell populations obtained from either the HIF-1α- or HIF-2α-depleted cells formed almost no tumors, as shown in Figure 6B. Taken together, these results clearly indicated that both HIF-1α and HIF-2α play key roles in promoting the *in vivo* aggressiveness and tumor growth of both CD44^+^ and CD44^−^/CD133^−^ subpopulations isolated from SW480 colon cancer cells, which exhibit constitutively active Wnt signaling. Therefore, these results suggest that HIFs may promote tumor growth and progression in cells with altered Wnt signaling in a manner that apparently does not depend on the expression of the CD44 stem cell marker.

### HIF-1α and HIF-2α knockdown produced opposing effects in canonical Wnt signaling

**Figure 7:**
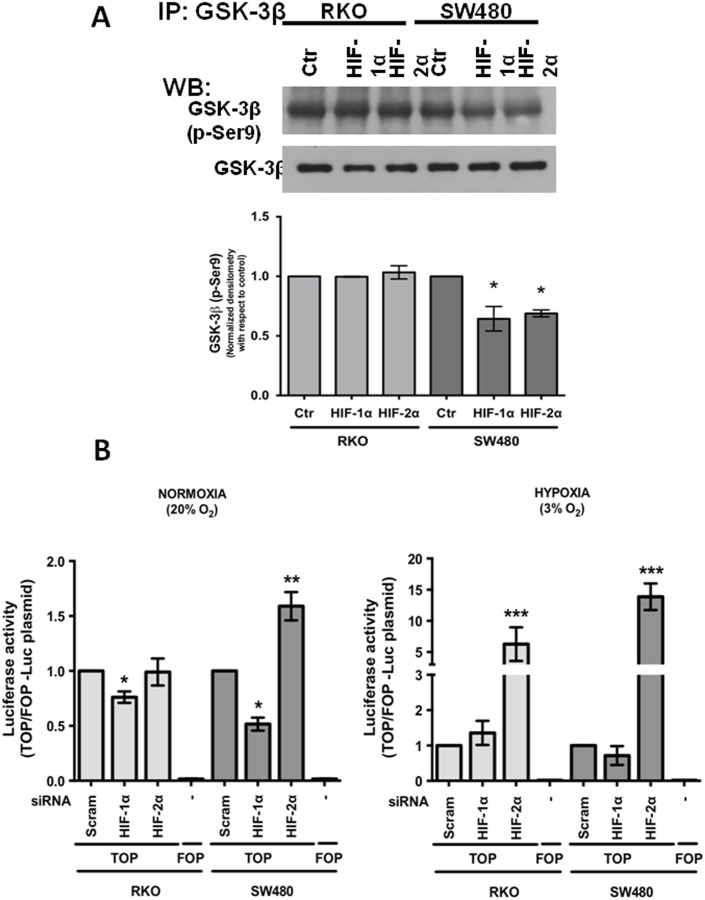
HIF-1α and HIF-2α knockdown produced opposite effects in canonical Wnt signaling. **A)** Stable HIF-1α- or HIF-2α-depleted cells exhibit decreased levels of the inactive (p-Ser9)-GSK-3β form. Stable control (scrambled shRNA, Ctr in the figure) or HIF-1α- or HIF-2α-silenced RKO or SW480 colon cancer cells were cultured under normoxia. Total cell extracts of the colon cell lines were prepared, and the samples were subjected to 10% SDS-PAGE and transferred onto nitrocellulose membranes. An immunoblot analysis was performed using anti-GSK-3β or anti-(p-Ser9)-GSK-3β, as indicated in the figure, and developed using a horseradish peroxidase-conjugated second antibody. Densitometric analysis was performed to estimate the level of (p-Ser9)-GSK-3β with respect to the level of total GSK-3β. All of the assays were performed in triplicate, and the data represent the means ± SEM from at least three independent assays. *: p<0.05. **B)** Stable control (scrambled shRNA, “Scram” in the figure) or HIF-1α- or HIF-2α-silenced SW480 or RKO cells were transiently transfected with the pTOPFlash or pFOPFlash (control) reporter plasmids. At 24 h post-transfection, the cells were incubated under normoxic (20% O_2_) or hypoxic conditions (3% O_2_) for 12 h. The cells were washed and lysed, and the luciferase activity was assayed. The activity was normalized with respect to the activity of *Renilla* luciferase or with respect to the protein content in each sample. All of the assays were performed in quintuplicate, and the data represent the means ± SEM from at least five independent assays. *: p<0.05; **: p<0.01; ***: p<0.001.

**Figure 8:**
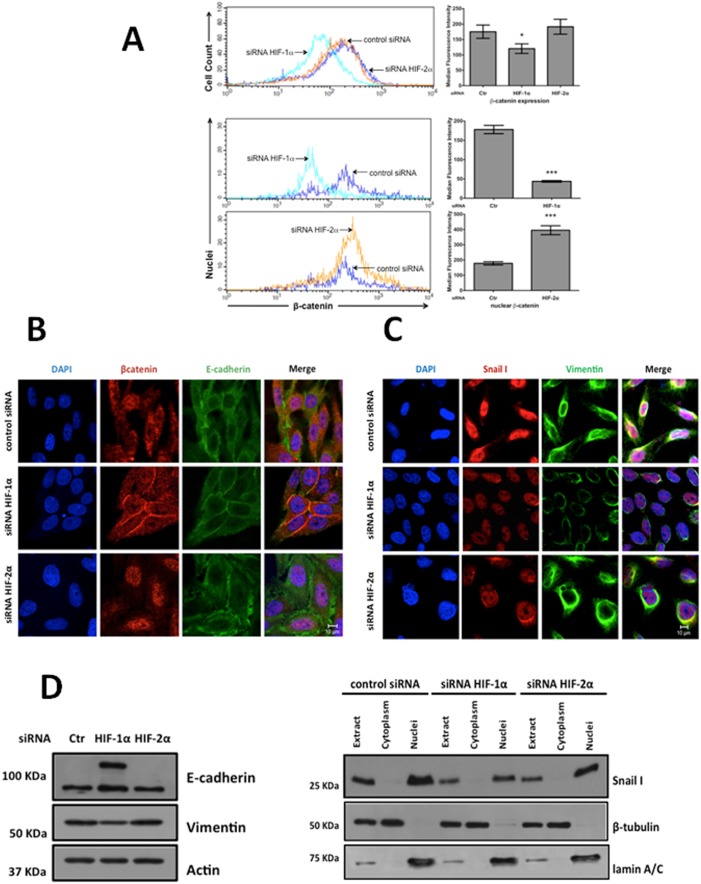
Stable knockdown of HIF-1α in SW480 cells results in decreased β-catenin expression levels and co-localization of β-catenin with E-cadherin at the cell membrane. **A)** Upper panel: Decreased β-catenin expression in HIF-1α-knockdown SW480 cells. The cells were incubated in the presence of EDTA, washed, and incubated with mouse anti-β-catenin antibody. The cells were washed and stained with goat Alexa647-conjugated anti-mouse secondary antibody and examined by flow cytometry. Controls stained with only the secondary antibody were prepared and were used as controls (not shown). The figure shows the overlapping histograms of the labeled SW480 control cells (blue line and Ctr in the bar graph), HIF-1α-silenced cells (green line), and HIF-2α-knockdown cells (orange line). The bar graph on the right shows the means of the median fluorescence intensity ± SEM from at least four independent experiments. *: p<0.05. **Middle panel:** Quantification of β-catenin in nuclei isolated from SW480 control (scrambled shRNA) or HIF-1α-depleted cells. **Lower panel:** Quantification of β-catenin in nuclei isolated from SW480 control (scrambled shRNA) or HIF-2α-depleted cells. The nuclei were purified and labeled with anti-β-catenin and with Alexa 647-conjugated goat anti-mouse antibody in staining buffer as described in the “Materials and Methods”. After the final wash, the nuclei were fixed and analyzed by flow cytometry. Independent gates were generated for intact cells and for isolated nuclei stained with propidium iodide prior to the flow cytometry analysis. The figure shows the overlapping histograms of the labeled SW480 control cells (blue line and Ctr in the bar graph), HIF-1α-knockdown cells (green line), and HIF-2α-knockdown cells (orange line). A representative histogram from at least three independent experiments is shown. *: p<0.05; ***: p<0.001. **B)** and **C)** Stable control (scrambled shRNA) or HIF-1α- or HIF-2α- silenced SW480 cells were fixed, permeabilized, and co-immunostained with antibodies against β-catenin and E-cadherin (panel B) or against Snail 1 and vimentin (panel C). Nuclear staining was obtained by incubating the coverslips with 4′6-diamidino-2-phenylindol (DAPI). The fluorescence was analyzed by laser confocal microscopy as described in the “Materials and Methods”. E-cadherin and vimentin were visualized with Cy3-conjugated goat anti-rabbit antibody, and β-catenin and Snail 1 were visualized with Alexa 647-conjugated goat anti-mouse antibody. Controls were stained only with the secondary antibody, and no fluorescence signals were obtained from these controls (not shown). The data are representative of three independent experiments. Scale bar: 10 µm. D) Expression analysis of the EMT-related markers by western blot. Cytoplasmic and nuclear fractions were obtained from the cell extracts of control (Ctr in the figure) or HIF-1α- or HIF-2α- silenced SW480 cells using a nuclear isolation kit (Sigma), and the presence of the proteins indicated in the figure was analyzed in each fraction and in cell extracts by western blot. β-tubulin and lamin A/C were employed as a cytoplasmic marker and a nuclear marker, respectively, to confirm a lack of contamination and thus successful fractionation. The data are representative of three independent experiments.

As previously mentioned, HIF-1α modulates Wnt/β-catenin in hypoxic embryonic stem cells by enhancing β-catenin activation and the expression of the downstream effectors LEF-1 and TCF-1 [11]. In contrast, the stability of HIF-1α is directly regulated by GSK-3β-mediated phosphorylation: the inhibition or depletion of GSK-3β increases HIF-1α levels, whereas the overexpression of GSK-3β reduces HIF-1α levels [21], [22]. Consistent with this finding, we found that stable HIF-1α- or HIF-2α-depleted cells exhibit decreased levels of inactive (p-Ser9)-GSK-3β, as can be observed in Figure 7A. Because the majority of the current knowledge on HIF is derived from studies performed under hypoxic conditions and because HIF stabilization has also been observed under normoxic conditions such as in this study (Figure 1), we investigated the effects of HIF knockdown on β-catenin transcriptional activity under normoxic and hypoxic conditions using the pTOPFlash/pFOPFlash reporter system as described in the Materials and Methods. We also used SW480 cells, which have constitutively active β-catenin-mediated transcriptional activity, and compared them with RKO cells, which have normal Wnt signaling. Stable control (scrambled shRNA plasmid)-, HIF-1α-, or HIF-2α-silenced SW480 or RKO cells were transiently transfected with pTOPFlash or pFOPFlash (control) plasmids. At 24 h post-transfection, the cells were incubated under normoxic or hypoxic conditions for 12 h, and the reporter activity was examined as shown in Figure 7B. Under normoxia, HIF-1α depletion resulted in a significant decrease in β-catenin transcriptional activity in both RKO and SW480 cells; in contrast, HIF-2α depletion enhanced this transcriptional activity, but only significantly in SW480 cells. Under hypoxia, while the silencing of HIF-1α did not induce significant changes in β-catenin transcriptional activity in either RKO or SW480 cells, HIF-2α depletion resulted in a substantial increase in this transcriptional activity in both cell types (Figure 7B). To explain these antagonistic effects, we first analyzed the expression levels of β-catenin by flow cytometry in SW480 cells. Figure 8A (upper) shows that consistent with the negative effects observed on β-catenin transcriptional activity, β-catenin protein expression decreased as a result of HIF-1α knockdown compared with the control cells, but did not increase as a result of HIF2-α silencing. Because β-catenin is principally regulated through its stability and localization, we examined the presence of nuclear β-catenin as a result of HIF knockdown. The quantification of the β-catenin present in nuclei obtained from control or HIF-1α- or HIF-2α-depleted cells by flow cytometry clearly showed that HIF-1α- depleted cells have less β-catenin in their nuclei than HIF-2α- depleted cells, which instead exhibit increased β-catenin nuclear protein levels (Figure 8A lower panel). In addition, the immunofluorescence assays depicted in Figure 8B indicated that the β-catenin intracellular localization was different between the HIF-1α- and HIF-2α-silenced SW480 cells: the β-catenin was mainly localized outside the nucleus and co-localized with E-cadherin at the plasma membrane in the HIF-1α-silenced cells, whereas it was only located in the cell nucleus of the HIF-2α knockdown cells. In addition, HIF-1α depletion induced a return to the epithelial cell morphology, suggesting that HIF-1α silencing induced a reversal of the EMT phenotype. To confirm this finding, we examined the protein levels of EMT-related markers by immunofluorescence and western blot. Figures 8C and 8D show that the protein levels of the EMT markers vimentin and nuclear Snail 1 were diminished as a result of HIF-1α depletion, whereas the level of the epithelial marker E-cadherin concomitantly increased. In addition, as can be observed in Figures 8C and 8D, the localization of these markers changed mainly as a result of HIF-1α silencing. In the case of HIF-2α silencing, we observed similar protein levels of vimentin and E-cadherin as in the control cells; however, the nuclear expression of Snail 1 was decreased as a result of HIF-2α depletion. These results are consistent with previous reports that showed that HIF-1α activates canonical Wnt to promote an EMT program in cancer cells [13], [23] and also show that HIF-2α silencing consistently enhances canonical Wnt activation by inducing the nuclear accumulation of the transcriptional co-activator β-catenin in both cell types, particularly under hypoxic conditions, suggesting that this protein may negatively modulate canonical Wnt signaling.

### HIF-1α and HIF-2α knockdown exerted opposing effects on the expression of the stem cell markers CD44 and Oct4, but only HIF-1α depletion increased the expression of the differentiation marker CK20

**Figure 9:**
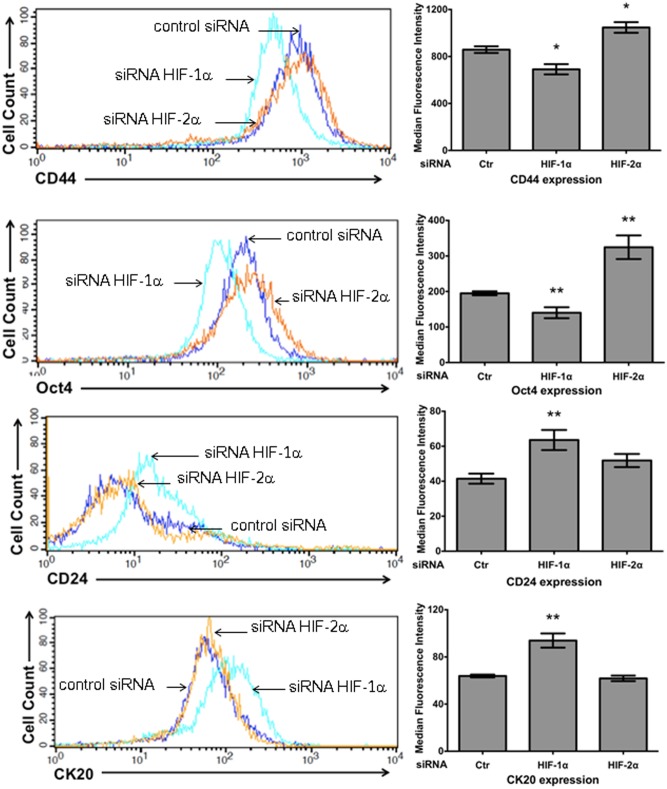
HIF-1α and HIF-2α knockdown exerted opposing effects on the expression of the stem cell markers CD44 and Oct4, but only HIF-1α depletion increased the expression of the differentiation marker CK20. Stable control (scrambled shRNA plasmid) or HIF-1α- or HIF-2α- silenced SW480 cells were incubated in the presence of EDTA, washed, and incubated with mouse anti-CD44, rabbit anti-Oct4, mouse anti-CD24 or mouse anti-CK20 antibodies. The cells were washed, stained with Alexa 647-conjugated goat anti-mouse/rabbit secondary antibody, and examined by flow cytometry. The figure shows the overlapping histograms of labeled SW480 control cells (blue line and Ctr in each bar graph), HIF-1α-knockdown cells (green line), and HIF-2α-knockdown cells (orange line). A representative histogram of the data is shown, and the bar graphs represent the means of the median fluorescence intensity ± SEM from at least four independent experiments. *: p<0.05; **: p<0.01.

We then examined the effect of HIF-1α or HIF-2α depletion on the expression profiles of stem cell markers by flow cytometry. SW480 cells have been reported to express CD44, with a subpopulation of these cells also expressing CD24 [24], reported to be a stem cell marker and adhesion molecule. In addition, HIFs have been reported to induce the gene expression signatures characteristic of human embryonic stem cells in aggressive tumors [25], including Oct4 and Sox2. The presence of the CD44 and Oct4 stem cell markers and the possible presence of CD24 and CK20, which has been widely used as an epithelial differentiation marker, were then analyzed by FACS. The results showed that the depletion of HIF-1α decreased the expression of CD44 and Oct4 and increased the expression of CD24 and CK20 with respect to the controls (Figure 9). In marked contrast to HIF-1α, HIF-2α knockdown did not affect the expression of CD24 and CK20 but did increase the expression of the stem cell markers CD44 and Oct4 compared with the controls. These findings are consistent with the hypotheses that HIF-1α participates in the induction of an EMT genetic program that allows cancer cells to acquire features of mesenchymal-like cells, and importantly, that HIF-1α and HIF-2α do not activate the same pathways to regulate stem cell maintenance or differentiation, arguing that these proteins play complementary and non-redundant roles in tumor biology.

## Discussion

HIFs play key roles in many crucial aspects of cancer biology including angiogenesis, stem cell maintenance, metabolic reprogramming, autocrine growth factor signaling, the EMT program, invasion and metastasis [7], [26]. Consistent with these functions, increased HIF-1α and HIF-2α protein expression has been observed in a broad array of human cancer cell types, and has been associated with poor prognosis in many cases. Importantly, high levels of expression have also been detected in tumor cells in the absence of hypoxia because elevated oncogenic signaling in cancer cells can induce HIF-α expression through O_2_-independent mechanisms, including increased transcription and/or translation of HIF-α mRNA [27]. In this respect, we also showed that only colon carcinoma cells co-express HIF-1α and HIF-2α under normoxic conditions, in contrast to non-malignant colon cells, which do not express these factors under these conditions.

In this study, we analyzed the participation of HIF-1α and HIF-2α in maintaining the malignant phenotype and in β-catenin-mediated transcriptional activity. We used two colon cancer cell lines that are representative of two different Wnt genetic contexts: RKO cells, which express a normal APC and display normal canonical Wnt signaling that depends on the ligand for activation, and SW480 cells, which only express mutant truncated APC and are representative of colon cancer cells that display constitutively active canonical Wnt signaling [16]. The stable silencing of HIF-1α or HIF-2α was induced by siRNA, and the effects of the depletion of these proteins were evaluated in Wnt/β-catenin signaling, in malignant phenotype maintenance, and in the expression of stem and differentiation markers under both normoxia and hypoxia. Although the silencing of either HIF-1α or HIF-2α negatively affected apoptosis resistance, tumor metabolism, migration, and the tumorigenic activity of several colon cancer cell lines, HIF-1α and HIF-2α deficiency also revealed isoform-specific effects on tumor cells, reinforcing the emerging concept that these two HIF isoforms can act antagonistically and not redundantly to regulate biological processes in malignancy [28]. In this regard, our data are in agreement with the results reported by Imamura et al. in colon cancer [10]. These researchers also demonstrated divergent cellular functions for HIF-α isoforms in SW480 colon cancer cells: the selective knockdown of HIF-1α resulted in lower rates of proliferation and migration *in vitro*, whereas the selective knockdown of HIF-2α exerted no effect on cellular proliferation *in vitro* but doubled the colony formation in soft agar assays [10].

With respect to the shared negative effects on apoptosis, lactate production, and migration observed in the present study as a result of the silencing of HIF-1α or HIF-2α, HIF-1α led to more severe negative effects compared with HIF-2α depletion. Although both factors share common downstream transcriptional targets such as vascular endothelial growth factor, each HIFα isoform also has unique targets. Analyses using high-resolution ChiP-seq techniques have revealed that HIF-1α and HIF-2α bind preferentially to specific genes [26]. For example, HIF-1α exhibits a significantly higher level of binding with glycolytic pathway genes, whereas HIF-2α binding exhibits greater binding with genes involved in invasion such as matrix metalloproteinases [29], PAI-1 [30], and the stem cell factor Oct-3/4 [9]. In agreement with this finding, and with previous reports that showed that HIF-1α plays an important role in glycolysis, autophagy, and apoptosis, our data showed that lactate secretion under normoxic conditions decreased by 50% as a result of HIF-1α silencing, whereas it only decreased by 20% as a result of HIF-2α depletion (Figure 2B). A similar phenomenon was observed with the apoptosis resistance of SW480 cells: a higher proportion of apoptosis was achieved by the blockade of HIF-1α expression than HIF-2α silencing with respect to the controls, and this difference was more evident when the cells were subjected to oxidative stress (see Figure 3). With respect to the effects of HIF silencing on migration, although the depletion of either HIF-1α or HIF-2α caused the inhibition of *in vitro* cell motility, the molecular mechanisms employed by each isoform appear to be different because HIF-1α silencing decreased CXCR4 expression. Consistent with this hypothesis, the induction of CXCR4 by hypoxia has been reported to depend on both the activation of HIF-1α and the stabilization of the transcript in cancer cells, which is paralleled by an increase in the chemotactic responsiveness to its specific ligand, SDF-1α [31].

The tumorigenic activity of the CD44^+^ and CD44^−^/CD133^−^ subpopulations isolated from SW480 control or HIF-silenced cells and the tumorigenic activity of metastatic SW620 cells were markedly negatively affected, but more importantly, the tumorigenic activities were similarly abolished as a result of the silencing of either HIF-1α or HIF-2α, emphasizing the importance of both factors in the promotion of tumor growth and progression *in vivo*. However, HIF-1α and HIF-2α knockdown produced opposite effects on canonical Wnt signaling and on the expression of cancer stem cell and differentiation markers. Although some previous studies have implicated HIF factors in Wnt/β-catenin signaling, the molecular mechanisms involved in their crosstalk remain poorly understood. We found that HIF-1α silencing decreases β-catenin transcriptional activity, whereas HIF-2α depletion greatly enhances this activity, particularly under hypoxic conditions. We also found that these opposing effects on Wnt activation can be explained by the finding that HIF-1α depletion not only decreases the β-catenin protein levels in cells and decreases the nuclear β-catenin levels but also induces the sequestration of β-catenin at the cell membrane by E-cadherin. The overall result is that β-catenin transcriptional activity is decreased. In marked contrast, we found that the enhancement of canonical Wnt activation upon HIF-2α silencing is mainly due to the induction of nuclear β-catenin accumulation, which suggests that HIF-2α negatively modulates canonical Wnt signaling. The molecular mechanism through which HIF-2α induces this effect remains to be elucidated. However, it is well established that the enhanced phosphorylation of the tyrosine residues on β-catenin is nearly invariably associated with its dissociation from adhesion complexes while enhancing BCL9-2 binding to promote transcription [32]–[34]. Interestingly, Franovic et al. [35] reported the unexpected observation that genetically diverse cancers converge at a common and obligatory growth axis instigated by HIF-2α. These researchers demonstrated that the inhibition of HIF-2α prevents the *in vivo* growth and tumorigenesis of highly aggressive glioblastoma, colorectal, and non-small-cell lung carcinomas and the *in vitro* autonomous proliferation of several other cancers, regardless of their mutational status and tissue of origin. These authors also showed that the concomitant deactivation of select receptor tyrosine kinases, including EGFR and IGF1R, as well as downstream ERK/Akt signaling, suggests that HIF-2α exerts its proliferative effects through these major pathways. Consistent with this finding, silencing these receptors results in the same phenotype as the loss of HIF-2α oncogenic activity [35].

During the progression to metastatic competence, carcinoma cells have been described as entering into an EMT program that allows them to acquire features of mesenchymal-like cells which may significantly favor invasiveness, including changes in adhesive properties, motility activation and the ability to degrade/remodel the extracellular matrix [12], [13], [23]. Both HIF-1α and canonical Wnt signaling have been reported to induce the EMT in cancer cells. Cannito et al. [12] provided evidence that hypoxia-dependent changes occur through a biphasic mechanism involving a very early and reactive oxygen species (ROS)-dependent inhibition of GSK-3β followed by early Snail 1 nuclear translocation, also involving the nuclear translocation of β-catenin. Other researchers have shown that the hypoxia-induced EMT may be enhanced by the addition of recombinant canonical Wnt3a, whereas it is repressed by β-catenin siRNA in hepatocellular carcinoma [13]. In addition, Zhao et al. [23] have found that the inhibition of Wnt signaling activity through β-catenin shRNA causes a reversal of the EMT induced by HIF-1α in human prostate cancer. In agreement with these findings, we observed that the depletion of HIF-1α decreased the expression of the stem cell markers CD44 and Oct4 compared with the controls and induced a reversal of the EMT phenotype, increasing the expression of the E-cadherin and CK20 epithelial markers and decreasing the expression of vimentin and nuclear Snail 1. In addition, silencing HIF-1α induced a decrease in β-catenin protein levels and its recruitment by E-cadherin into the cell membrane, preventing its nuclear localization and transactivation. In marked contrast, the blockade of HIF-2α expression produced the opposite effects on the expression of stem cell markers and did not induce the expression of epithelial markers.

Taken together, our results reveal complex roles for HIF-1α and HIF-2α in colon cancer cells, as has also been demonstrated in other cell types. Given the disparate effects that HIF-1α and HIF-2α may induce on tumor growth and progression, it will be critical to determine whether potential HIF inhibitors affect both HIF-α subunits equally, particularly in cancers that express both HIF-1α and HIF-2α, such as colon cancer, where these isoforms appear to play distinct roles.

## Supporting Information

Figure S1 **FACS analysis of HIF-1α or HIF-2α expression levels in SW480 cells cultured under normoxic or hypoxic conditions.** Stable control (scrambled shRNA) or HIF-1α- or HIF-2α- silenced SW480 cells were incubated in the presence of EDTA, washed, and incubated with mouse anti-HIF-1α or anti-HIF-2α. The cells were washed, stained with Alexa647-conjugated goat anti-mouse secondary antibody and examined by flow cytometry. The levels of HIF-1α and HIF-2α expression shown in bar graphs were estimated by normalizing to the corresponding expression levels observed in control cells. All of the assays were performed in triplicate, and the data represent the means ± SEM from at least three independent assays. *: p<0.05.
(TIF)
