# Supplementary figures and images for: Hypoxia-Inducible Factors Modulate the Stemness and Malignancy of Colon Cancer Cells by Playing Opposite Roles in Canonical Wnt Signaling
Source: PLoS One. 2014 Nov 14;9(11):e112580. doi: 10.1371/journal.pone.0112580 (PMC4232394; doi:10.1371/journal.pone.0112580)

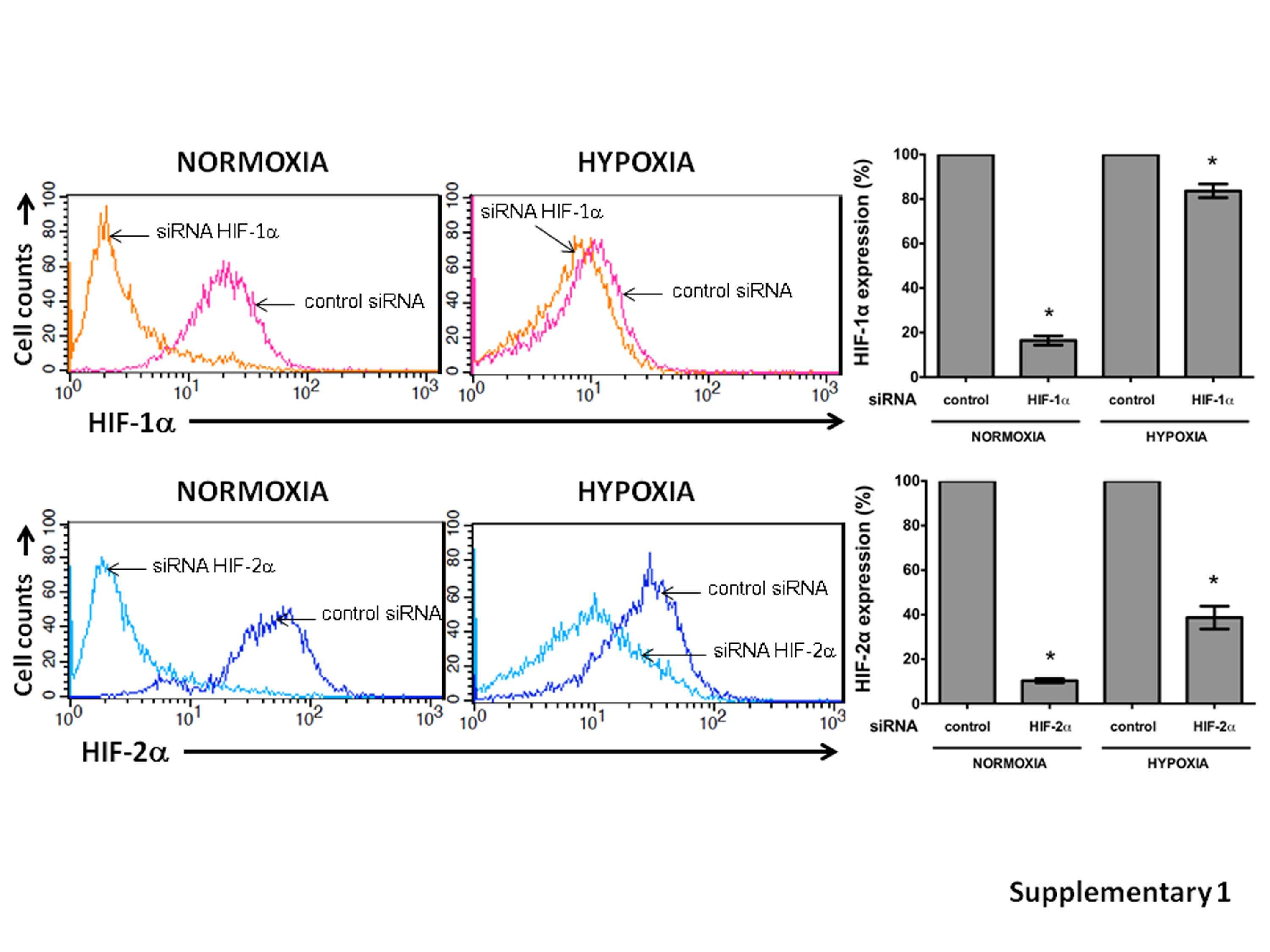

Supplement: Figure S1 — FACS analysis of HIF-1α or HIF-2α expression levels in SW480 cells cultured under normoxic or hypoxic conditions. Stable control (scrambled shRNA) or HIF-1α- or HIF-2α- silenced SW480 cells were incubated in the presence of EDTA, washed, and incubated with mouse anti-HIF-1α or anti-HIF-2α. The cells were washed, stained with Alexa647-conjugated goat anti-mouse secondary antibody and examined by flow cytometry. The levels of HIF-1α and HIF-2α expression shown in bar graphs were estimated by normalizing to the corresponding expression levels observed in control cells. All of the assays were performed in triplicate, and the data represent the means ± SEM from at least three independent assays. *: p<0.05. (TIF) [file pone.0112580.s001.tif]
